# Liproxstatin-1 Attenuates Retinal Ischemia–Reperfusion Injury by Suppressing EGR1-Mediated Ferroptosis

**DOI:** 10.3390/antiox15030391

**Published:** 2026-03-19

**Authors:** Wei Huang, Yue Dong, Xuan Zhou, Huishan Lin, Jingwei Yao, Zhuoyi Wu, Weng Ian Tam, Yuheng Tan, Chengguo Zuo, Mingkai Lin

**Affiliations:** State Key Laboratory of Ophthalmology, Zhongshan Ophthalmic Center, Sun Yat-sen University, Guangdong Provincial Key Laboratory of Ophthalmology Visual Science, Guangzhou 510060, Chinawuzhy233@mail2.sysu.edu.cn (Z.W.); tanyh33@mail2.sysu.edu.cn (Y.T.)

**Keywords:** ferroptosis, liproxstatin-1, retinal ischemia–reperfusion, retinal ganglion cells, EGR1/p53/xCT pathway

## Abstract

Retinal ischemia–reperfusion (I/R) injury results in irreversible vision loss largely through retinal ganglion cell (RGC) death, with ferroptosis being a key mechanism. This study evaluated the therapeutic potential of the ferroptosis inhibitor Liproxstatin-1 (Lip-1) and deciphered its underlying mechanism. Using a mouse retinal I/R model and primary RGC cultures subjected to oxygen–glucose deprivation/reoxygenation (OGD/R), we demonstrated that Lip-1 effectively inhibits ferroptosis. Lip-1 treatment preserved retinal architecture (as assessed by H&E staining and SD-OCT) and partially restored visual function (as measured by electroretinography). Integrated molecular analyses—including immunofluorescence, Western blotting, and RNA sequencing—showed that Lip-1 downregulates early growth response 1 (EGR1), thereby inhibiting p53 and consequently restoring solute carrier family 7 member 11 (xCT) expression. Crucially, lentivirus-mediated EGR1 knockdown attenuated OGD/R-induced ferroptosis, confirming its pivotal role. Our work defines a coherent EGR1–p53–xCT signaling axis driving ferroptosis in retinal I/R injury and identifies Lip-1 as a neuroprotective agent targeting this pathway. These findings establish a druggable ferroptotic cascade and provide a mechanistic rationale for targeting EGR1 in the treatment of ischemic retinopathies.

## 1. Introduction

Glaucoma is a leading cause of irreversible blindness, projected to affect 112 million individuals by 2040 [1,2]. This neurodegenerative disorder is characterized by progressive retinal ganglion cell (RGC) loss and optic nerve degeneration [1,2]. Although intraocular pressure (IOP) reduction remains the mainstay of therapy [3], a substantial subset of patients continue to decline despite well-controlled IOP [4], implicating pressure-independent mechanisms in RGC demise.

Retinal ischemia–reperfusion (I/R) injury—a well-established experimental paradigm that mirrors the microvascular compromise observed in glaucoma patients [5]—actively promotes RGC death via oxidative stress, mitochondrial dysfunction, and excitotoxic signaling [6,7]. Clinical evidence increasingly reinforces this connection; individuals with glaucoma exhibit significantly lower macular superficial vascular density and flow area compared to healthy controls, with these microvascular deficits correlating robustly with both structural thinning and functional loss [8]. These findings transform I/R injury from a theoretical contributor into a measurable, targetable pathological event in human glaucoma, underscoring the urgency of dissecting its downstream executioner mechanisms.

Ferroptosis—an iron-dependent cell death program triggered by unrestrained lipid peroxidation [9]—has now emerged as a key mechanistic link between microvascular injury and glaucomatous neurodegeneration [10,11]. Its hallmarks—glutathione depletion, GPX4 inactivation, ACSL4-mediated polyunsaturated fatty acid peroxidation, and distinctive mitochondrial condensation [12,13]—have been documented in RGCs from human glaucomatous retinas [14,15]. Experimentally, retinal I/R injury disrupts iron homeostasis and triggers ferroptosis within hours, preceding other cell death pathways [16,17,18]. Pharmacological inhibition with ferrostatin-1 (Fer-1) or melatonin confers robust RGC protection [15,19], validating ferroptosis as a druggable target in glaucoma. However, these agents act primarily as non-specific radical-trapping antioxidants, offering limited insight into upstream regulatory nodes—a limitation that underscores the need for next-generation ferroptosis inhibitors with defined mechanistic targets.

Liproxstatin-1 (Lip-1) is a potent, radical-trapping ferroptosis inhibitor [20]. Its reactive -NH group donates a hydrogen atom to lipid peroxyl radicals (LOO•), and its molecular conformation enables efficient integration into lipid bilayers, creating a localized antioxidant microenvironment [20]. Notably, oxidation products of Lip-1 retain radical-trapping activity with higher stoichiometry (n-value) than conventional antioxidants, affording sustained protection [20]. While Lip-1 attenuates ferroptosis in I/R models of multiple organs [21,22], its role in retinal I/R injury remains undefined. In particular, whether Lip-1 engages transcriptional programs upstream of ferroptotic execution remains unexplored—a gap that underscores the need to identify context-specific transcriptional drivers of RGC ferroptosis. One such driver has recently emerged: the transcription factor early growth response protein 1 (Egr1) [23].

Egr1 is rapidly and robustly upregulated in response to I/R injury across multiple organs, including the heart, kidney, and lung, where it acts as a master transcriptional switch that amplifies inflammatory cascades and exacerbates oxidative stress [23,24,25,26]. Genetic ablation or targeted silencing of Egr1 confers marked protection against I/R-induced tissue damage, as reflected by reduced infarct size, suppressed pro-inflammatory cytokine expression, and preserved organ function [24,26,27]. Notably, Egr1 promotes ferroptosis through direct activation of pro-ferroptotic gene programs that disrupt redox homeostasis and drive iron-dependent lipid peroxidation [28,29]. Lip-1 has been shown to attenuate renal I/R injury through specific interference with Egr1-driven transcriptional networks, rather than through broad antioxidant effects alone [23]. Whether this transcriptionally targeted mechanism operates in the retina, however, remains entirely unexplored. Furthermore, the specific downstream signaling axis through which Egr1 might regulate ferroptosis in RGCs—such as the putative p53/xCT pathway [23]—has yet to be elucidated. Thus, investigating Lip-1 in the context of retinal I/R injury is not merely a test of another ferroptosis inhibitor; it represents a hypothesis-driven interrogation of a pathway-specific, transcriptionally anchored intervention within the ferroptotic cascade.

To address these gaps, this study aimed to investigate the therapeutic potential of Lip-1 in preserving retinal structure and function by inhibiting RGC ferroptosis, to identify the key signaling pathway responsible for its protective effects, and to mechanistically validate this pathway using genetic knockdown. Utilizing an in vivo murine retinal I/R model and an in vitro system of primary RGCs subjected to oxygen–glucose deprivation/reoxygenation (OGD/R), we sought to determine whether Lip-1 could attenuate RGC ferroptosis, and to explore the potential involvement of the EGR1-related signaling axis in this process.

## 2. Materials and Methods

### 2.1. Animals and Experimental Design

Male C57BL/6J mice (6–8 weeks, 20–25 g; GemPharmatech, Nanjing, China) were housed under specific pathogen-free conditions (22 ± 1 °C, 55 ± 5% humidity, 12 h light/dark cycle) with free access to autoclaved food and water. All procedures adhered to the ARVO Statement for the Use of Animals in Ophthalmic and Vision Research and the Chinese National Standard GB/T 35892-2018 (Standardization Administration of China, Beijing, China) and were approved by the Institutional Animal Care and Use Committee of Zhongshan Ophthalmic Center (Approval No. O2023020).

A total of 120 mice (240 eyes) were used in this study, with sample sizes predetermined based on pilot experiments and power analysis to ensure adequate statistical power for each assay. Eyes were randomly divided into four groups by an independent researcher using complete randomization: a sham + vehicle (sham) group, a sham + Lip-1 (Lip-1) group, an I/R + vehicle (I/R) group, and an I/R + Lip-1 (I/R + Lip-1) group. For dose-response analysis, separate cohorts received Lip-1 at concentrations of 50, 100, 200, 500, or 1000 nM or vehicle prior to I/R (*n* = 6 eyes per concentration), establishing 200 nM as the optimal dose for subsequent experiments based on retinal wholemount immunostaining for RGC markers (Tuj1 and RBPMS) at 7 days post-I/R. For day 7 post-I/R analyses, a cohort of eyes from each main group (*n* = 12 eyes per group) was used, with 6 eyes per group processed for SD-OCT followed by H&E staining, and the remaining 6 eyes per group used for ERG followed by immunofluorescence staining of retinal cryosections. For day 1 post-I/R analyses, another cohort from each main group (*n* = 18 eyes per group) was employed, allocating 6 eyes per group for immunofluorescence assessment of ferroptosis markers on retinal cryosections, 6 eyes per group for immunofluorescence evaluation of signaling pathway components, and 6 eyes per group for Western blot analysis (3 eyes for ferroptosis markers and 3 eyes for signaling pathway validation). For RNA sequencing at day 1 post-I/R, an independent cohort comprising three groups (Sham, I/R, and I/R + Lip-1) was included with four biological replicates per group, each replicate consisting of pooled retinal tissue from six eyes (totaling 72 eyes, 24 eyes per group).

Throughout the study, group allocations were concealed from all investigators involved in surgeries, treatment administration, outcome assessment, and data analysis until the study endpoint; animal health was monitored at least twice daily with predefined humane endpoints (>20% body weight loss, inability to access food or water, or severe distress), and no animals met these criteria. For statistical comparisons, each group was compared with the Sham group to assess baseline effects, whereas the I/R + Lip-1 group was specifically compared to the I/R group to evaluate the therapeutic effect of Lip-1.

### 2.2. Retinal I/R Model

Retinal I/R injury was induced by anterior chamber cannulation [30]. After anesthesia with pentobarbital sodium (50 mg/kg, i.p.), mice received topical tetracaine (0.5%) and tropicamide phenylephrine (1%) for corneal anesthesia and pupil dilation. A 32-gauge needle connected to a saline reservoir was inserted into the anterior chamber to maintain IOP at 110 mmHg for 60 min. Contralateral eyes received sham surgery without IOP elevation. Eyes with aqueous leakage or lens damage were excluded. Tobramycin ointment was applied postoperatively before recovery at 37 °C.

### 2.3. Drug Administration

Lip-1 (Selleckchem, #S7699) was dissolved in dimethyl sulfoxide (DMSO; Solarbio, #D8371, Beijing, China) to generate a 5 mM stock solution, aliquoted, and stored light-protected at −80 °C. Before use, working concentrations (50–1000 nM) were prepared in sterile PBS (Servicebio, #G4202, Wuhan, China) and administered by intravitreal injection (2 µL/eye) 30 min prior to I/R induction. Vehicle (0.1% DMSO in PBS) was applied similarly.

### 2.4. Electroretinography (ERG)

Full-field ERGs were recorded from dark-adapted mice at 7 days post-I/R, following a previously described protocol [31]. Mice were prepared as detailed in Section 2.2. Corneal electrodes were placed using hydroxypropyl methylcellulose gel to ensure impedance remained below 7 kΩ. Photopic ERG responses were acquired at flash intensities of 0.01, 3.0, and 10 cd·s/m^2^ using a RETIscan^®^ system. The b-wave amplitude was quantified across all intensities, while the a-wave, photopic negative response (PhNR), and oscillatory potentials (OPs) were analyzed at 3.0 and 10 cd·s/m^2^.

### 2.5. Spectral-Domain Optical Coherence Tomography (SD-OCT)

SD-OCT was performed as previously described [32]. Briefly, at 7 days post-I/R, anesthetized mice were subjected to retinal imaging using a Heidelberg Spectralis SD-OCT device equipped with a 25° lens. Following pupil dilation and induction of corneal anesthesia, scans were acquired at a distance of 0.42 mm from the optic disc. The thickness of the total retina, retinal nerve fiber layer (RNFL), and ganglion cell complex (GCC) was then measured.

### 2.6. Hematoxylin and Eosin (H&E) Staining

Following transcardial PBS perfusion, enucleated eyes were fixed in FAS eyeball fixative (Servicebio, #G1109; 24 h, 4 °C), dehydrated through a graded ethanol series, and paraffin-embedded. Serial sagittal sections (8 µm thickness) passing through the optic disc were stained with hematoxylin and eosin (H&E). Histological images were acquired using a Pannoramic MIDI slide scanner (3DHISTECH, Budapest, Hungary) at 40× magnification. The thickness of retinal layers—including the nerve fiber layer/ganglion cell layer (NFL/GCL), inner plexiform layer (IPL), inner nuclear layer (INL), outer plexiform layer (OPL), and outer nuclear layer (ONL)—was measured using ImageJ software (version 1.54p, National Institutes of Health, NIH, Bethesda, MD, USA).

### 2.7. RNA Sequencing and Bioinformatic Analysis

Retinas from Sham, I/R, and I/R + Lip-1 groups were rinsed in ice-cold PBS, flash-frozen in liquid nitrogen, and processed by Shanghai Applied Protein Technology Co., Ltd. (Shanghai, China). for RNA sequencing. Differential expression analysis used limma–voom (R v4.2.1; thresholds: |log2FC| > 1.5, FDR-adjusted *p* < 0.05).

### 2.8. Western Blot (WB)

Retinal lysates were prepared using RIPA buffer with protease/phosphatase inhibitors. Proteins (20 μg per lane) were separated by SDS-PAGE, transferred to PVDF membranes, and blocked. Membranes were incubated with primary antibodies (Table 1) overnight at 4 °C, followed by HRP-conjugated secondary antibodies. After washing, signals were developed with ECL substrate and quantified using ImageJ.

### 2.9. Immunofluorescence (IF)

Retinal cryosections or whole-mounts were permeabilized with 0.3% Triton X-100, blocked in 10% normal goat serum, and incubated with primary antibodies (Table 1) overnight at 4 °C. After washing, samples were incubated with Alexa Fluor-conjugated secondary antibodies, counterstained with DAPI, and imaged using a Zeiss LSM 980 confocal microscope.

### 2.10. Primary RGC Culture, Lentiviral Transduction, and OGD/R Injury Induction

Primary RGCs were isolated from P0 neonatal mice via trypsin digestion and cultured in poly-D-lysine-coated dishes with Neurobasal-A medium supplemented with B27, GlutaMAX, and penicillin/streptomycin (all from Thermo Fisher Scientific, Waltham, MA, USA) [33]. For each independent biological replicate, retinas from a single litter were pooled and evenly distributed across experimental conditions. A minimum of three independent isolations from separate litters were performed for each experiment. OGD/R was induced in vitro on day 6 by 1 h incubation in glucose-free medium under hypoxic (1% O_2_) conditions, followed by 24 h reperfusion in complete medium under normoxia. Normoxic cultures served as controls.

To examine ferroptosis dynamics, cells were subjected to OGD/R for 3, 6, 12, or 24 h and compared to normoxic controls (n = 3 per group). For pharmacological inhibition, cells were pretreated with Lip-1 or vehicle prior to OGD/R. The effect of Lip-1 was assessed by comparing the OGD/R + Lip-1 group with the OGD/R + vehicle group, with parallel normoxic control groups (Control) establishing baselines.

For EGR1 knockdown, cells were transduced on day 2 with lentivirus carrying either a non-targeting control (shNC) or an EGR1-targeting (shEgr1) shRNA at MOI 10. Knockdown efficiency was validated across three independent shEgr1 constructs, each compared to shNC under OGD/R conditions. In subsequent functional assays, the OGD/R + shEgr1 group was compared to the OGD/R + shNC group to evaluate the impact of EGR1 knockdown, with corresponding normoxic controls (Control + shNC, Control + shEgr1).

All experimental allocations were performed using complete randomization by a blinded researcher.

### 2.11. Lipid Peroxidation Detection

Lipid peroxidation was assessed using BODIPY 581/591 C11 (GLPBIO, #GC40165, Montclair, CA, USA). Primary RGC cultures and retinal cryosections were incubated with 2 µM BODIPY^581/591^ C11 in PBS for 30 min at 37 °C under 5% CO_2_ in the dark. After washing with PBS, samples were fixed with 4% PFA for 30 min at room temperature. For cryosections, an additional air-drying step was included prior to imaging. Samples were mounted with anti-fade mounting medium and visualized by confocal microscopy (λex/em = 581/591 nm).

### 2.12. Mitochondrial Membrane Potential Assessment

Mitochondrial membrane potential was monitored using the JC-1 assay kit (Beyotime, #Y242679, Shanghai, China). Primary RGCs were incubated with 5 µg/mL JC-1 working solution, prepared according to the manufacturer’s instructions, for 30 min at 37 °C under 5% CO_2_ in the dark. Cells were then washed with PBS and immediately subjected to confocal microscopy (λex/em = 488/525 nm).

### 2.13. Cell Viability Assessment

Cell viability was determined using the CCK-8 kit (GLPBIO, #GK10001). Following 6-day differentiation in 96-well plates, RGCs were pretreated with Lip-1 (50–1000 nM) for 0.5 h prior to OGD/R induction. At 24 h post-reperfusion, 10 µL of CCK-8 solution was added to each well (n = 8 replicates per concentration) and incubated for 4 h at 37 °C under 5% CO_2_. Absorbance was measured at 450 nm using a microplate reader.

### 2.14. Labile Iron Pool Detection

The labile iron pool was detected using FerroOrange (Dojindo, #F374, Kumamoto, Japan). Primary RGCs and retinal cryosections were incubated with 1 µM FerroOrange in serum-free medium. Incubation was carried out for 30 min at 37 °C under 5% CO_2_ for cells, and at room temperature for cryosections. After incubation, samples were washed with PBS and imaged by confocal microscopy.

### 2.15. Superoxide Detection

Superoxide levels were detected using dihydroethidium (DHE; Thermo Fisher Scientific, #D11347, Waltham, MA, USA). Retinal cryosections were fixed in 4% PFA, permeabilized with 0.1% Triton X-100, and blocked with 5% donkey serum. After immunostaining, sections were incubated with 10 µM DHE in serum-free medium for 30 min at 37 °C in the dark. Nuclei were counterstained with DAPI, and images were acquired using a Zeiss LSM 980 confocal microscope.

### 2.16. Statistical Analysis

Data are presented as mean ± SEM from at least three independent biological replicates. Prior to analysis, normality was assessed using the Shapiro–Wilk test, and homogeneity of variances was examined using Levene’s test; all data met the assumptions for parametric analysis. For comparisons involving a single independent variable, statistical significance was determined using one-way ANOVA followed by Tukey’s post hoc test. For the experiment with two independent variables, two-way ANOVA was used to evaluate the main effects and interaction, followed by Tukey’s post hoc test for multiple comparisons. A *p*-value of less than 0.05 was considered statistically significant. All analyses were performed using GraphPad Prism 9.0.

## 3. Results

### 3.1. Lip-1 Pretreatment Attenuates Neuronal Cell Death in Retinal I/R Injury

**Figure 1:**
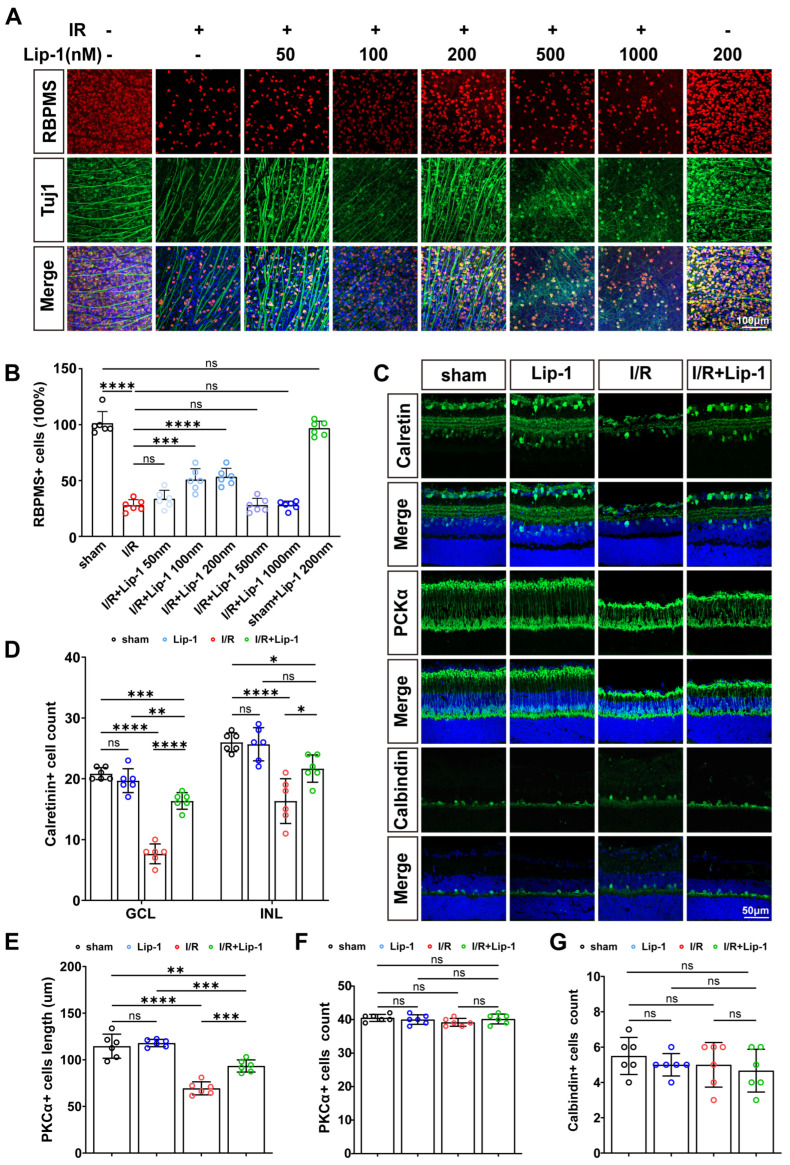
**Lip-1 attenuates neuronal cell death in retinal I/R injury.** (**A**) Retinal whole-mounts from I/R-injured mice intravitreally injected with Vehicle or Lip-1 (50, 100, 200, 500, or 1000 nM), immunostained for RBPMS (red), Tuj1 (green), and DAPI (blue) to label RGCs. Scale bar: 100 μm. Total magnification: 200× (**B**) Quantification of RBPMS-positive RGCs counts from (**A**) (*n* = 6 biologically independent experiments) Each colored circle represents an individual data point: sham (black), I/R + Vehicle (red), 50 nM Lip-1 (light blue), 100 nM Lip-1 (sky blue), 200 nM Lip-1 (deep sky blue), 500 nM Lip-1 (purple), 1000 nM Lip-1 (blue), and sham + 200 nM Lip-1 (green). (**C**) Retinal sections immunostained for calretinin (green), PKC-α (green), calbindin (green) and DAPI (blue) from sham and I/R mice treated with Vehicle or Lip-1. Scale bar: 50 μm. Total magnification: 400×. (**D**) Quantification of calretinin-positive cell counts in the GCL and INL from (**B**) (*n* = 6 biologically independent experiments). (**E**) Quantification of the total dendritic length of PKC-α-positive cells from (**B**) (*n* = 6 biologically independent experiments). (**F**) Quantification of PKC-α-positive cell counts from (**B**) (*n* = 6 biologically independent experiments). (**G**) Quantification of calbindin-positive cell counts from (**B**) (*n* = 6 biologically independent experiments). Data in panel (**B**) were analyzed by one-way ANOVA, and data in panels (**C**–**F**) by two-way ANOVA, both followed by Tukey’s post hoc test. All data are shown as mean ± SEM. ns, not significant, * *p* < 0.05, ** *p* < 0.01, *** *p* < 0.001, **** *p* < 0.0001.

To assess the therapeutic potential of ferroptosis inhibition in retinal I/R injury, Lip-1 was administered via intravitreal injection 0.5 h prior to the induction of ischemia. Immunostaining for the RGC-specific markers revealed a significant loss of RGCs in the I/R group compared with the sham-operated control (*p* < 0.0001), as assessed by retinal whole-mount labeling for neuron-specific class III beta-tubulin (Tuj-1) and RNA-binding protein with multiple splicing (RBPMS) (Figure 1A,B). Pretreatment with Lip-1 (50–1000 nM) significantly attenuated this neuronal loss in a dose-dependent manner, with maximal protective efficacy observed at 200 nM (*p* < 0.0001 vs. I/R). Higher concentrations (>500 nM) confer no additional survival benefit (*p* > 0.9999 vs. I/R), and no evidence of compound-related neurotoxicity was observed at the 200 nM dose (*p* = 0.9695 vs. sham) (Figure 1A,B).

**Figure 2:**
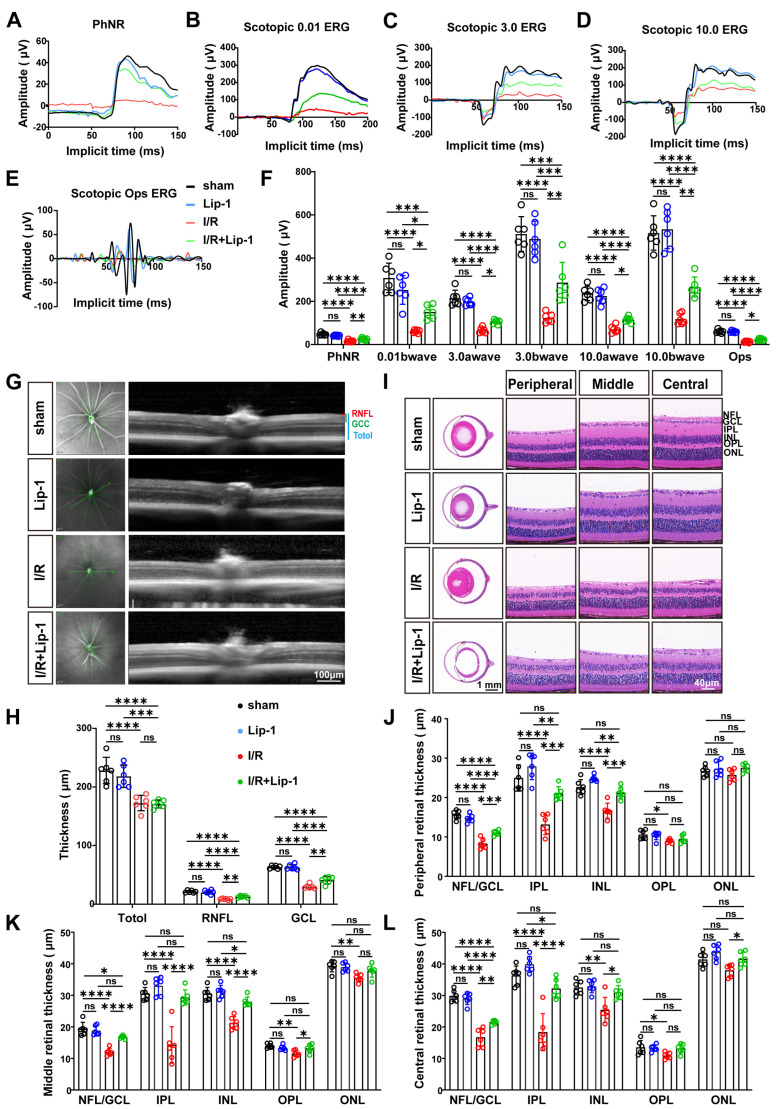
**Lip-1 Preserves Retinal Structure and Function in I/R Injury.** Representative PhNR (**A**), scotopic ERG (**B**–**D**), and OPs (**E**) waveforms from sham and I/R mice treated with Vehicle or Lip-1 under different flashlight intensities. (**F**) Quantification of PhNR amplitudes, a-wave amplitudes, b-wave amplitudes, and OPs amplitudes (*n* = 6 biologically independent experiments). (**G**) Representative high-resolution SD-OCT images of retinas from sham and I/R mice treated with Vehicle or Lip-1. Scale bars: 100 µm. (**H**) Quantification of RNFL, GCC, and total retinal thickness measured by high-resolution SD-OCT (*n* = 6 biologically independent experiments). (**I**) Representative HE staining images of paraffin-embedded retinal sections from sham and I/R mice treated with Vehicle or Lip-1. Scale bars: 40 µm (a white scale bar, total magnification: 400×), 1mm (a black scale bar, total magnification: 25×). Quantification of the thickness of the NFL/GCL, IPL, INL, OPL, and ONL in peripheral (**J**), middle (**K**), and central (**L**) retina regions from sham and I/R mice treated with Vehicle or Lip-1 (*n* = 6 biologically independent experiments). Data were analyzed by two-way ANOVA with Tukey’s post hoc test. All data are shown as mean ± SEM. ns, not significant, * *p* < 0.05, ** *p* < 0.01, *** *p* < 0.001, **** *p* < 0.0001. PhNR, photopic negative response; ERG, electroretinography; OPs, oscillatory potentials; SD-OCT, spectral-domain optical coherence tomography; RNFL, retinal nerve fiber layer; GCC, ganglion cell complex; HE, hematoxylin and eosin; Lip-1, Liproxstatin-1; NFL/GCL, nerve fiber layer/ganglion cell layer; IPL, inner plexiform layer; INL, inner nuclear layer; OPL, outer plexiform layer; ONL, outer nuclear layer.

Subtype-specific analyses further revealed the differential susceptibility of inner retinal neurons to I/R injury and their response to Lip-1 pretreatment. Calretinin-positive amacrine cells exhibited marked depletion in both the GCL (*p* < 0.0001 vs. sham) and INL (*p* < 0.0001 vs. sham) following I/R, which was significantly attenuated by Lip-1 pretreatment (*p* < 0.0001, *p* = 0.0120, respectively, vs. I/R) (Figure 1C,D). In PKCα-positive rod bipolar cells, I/R injury induced pronounced axonal truncation and simplification of dendritic arbors (*p* < 0.0001 vs. sham) without significant loss of somal integrity (*p* = 0.3055 vs. sham) (Figure 2C,E,F). These structural alterations were prevented by Lip-1 (*p* = 0.0004 vs. I/R) (Figure 1C,E,F). In contrast, calbindin-positive horizontal cells remained unaffected across groups (all *p* > 0.05) (Figure 1C,G).

### 3.2. Lip-1 Pretreatment Ameliorates I/R-Induced Retinal Impairments

To determine whether the observed neuroprotection translated into preservation of retinal function, ERG was performed under scotopic and photopic conditions following I/R injury with or without Lip-1 pretreatment. I/R injury induced profound functional deficits across multiple ERG parameters (Figure 2A–F). The PhNR, a specific correlate of RGC function, was markedly suppressed in the I/R group compared to the sham control (Figure 2A,F, *p* < 0.0001). Similarly, scotopic ERG revealed significant reductions in a-wave amplitudes at both 3.0 and 10.0 cd·s/m^2^ (both *p* < 0.0001, vs. sham) (Figure 2C,D,F). b-wave amplitudes were diminished at all flash intensities tested (0.01, 3.0, and 10.0 cd·s/m^2^; all *p* < 0.0001, vs. sham) (Figure 2B–D,F). OPs were also significantly attenuated following I/R (*p* < 0.0001, vs. sham) (Figure 2E,F). Lip-1 pretreatment significantly ameliorated these functional impairments. Notably, PhNR amplitudes showed significant improvement in the Lip-1-treated group relative to I/R alone (*p* = 0.0073) (Figure 2A,F), consistent with the RGC survival data. Furthermore, a-wave amplitudes were partially restored at both 3.0 and 10.0 cd·s/m^2^ (*p* =0.270, *p* = 0.0401, respectively, vs. I/R) (Figure 2C,D,F). b-wave responses showed significant recovery across all intensities (*p* = 0.0269 at 0.01, *p* = 0.0059 at 3.0 cd·s/m^2^, and *p* = 0.0043 at 10.0 cd·s/m^2^, vs. I/R). OPs amplitude were also partially rescued by Lip-1 (*p* = 0.0112, vs. I/R) (Figure 2E,F).

To determine whether functional preservation correlated with structural integrity, retinal morphology was assessed by H&E staining and SD-OCT (Figure 2G–L). SD-OCT analysis confirmed that I/R injury caused significant thinning of total retina, RNFL, and GCL (all *p* < 0.0001, vs. sham) (Figure 2G,H). Although total retinal thickness was not significantly improved by Lip-1 (*p* = 0.9982, vs. I/R), GCC and RNFL thickness were partially preserved (*p* = 0.0062 and *p* = 0.0011, respectively, vs. I/R) (Figure 2G,H). H&E staining confirmed these findings, showing marked I/R-induced thinning of inner retinal sublayer thickness (NFL/GCL, IPL, INL) (all *p* < 0.0001 vs. sham) (Figure 2I–L). In the outer retina, OPL thickness was uniformly reduced (peripheral, *p* = 0.0316; middle, *p* = 0.0020; central, *p* = 0.0345, vs. sham). In contrast, ONL thinning was confined to the middle region (*p* = 0.0044, vs. sham), with no significant changes in peripheral or central regions (peripheral, *p* = 0.6475; central, *p* = 0.0630, vs. sham). Lip-1 pretreatment significantly attenuated inner retinal thinning (all *p* < 0.05 vs. I/R) and improved ONL thickness in the central region (*p* = 0.0457, vs. I/R), but exerted no effect on OPL or ONL thickness in the middle and peripheral regions (all *p* > 0.05, vs. I/R) (Figure 2I–L). Lip-1 alone did not alter retinal structure or function compared to sham (all *p* > 0.05, vs. I/R).

### 3.3. Lip-1 Pretreatment Halts the Oxidative Stress-to-Ferroptosis Cascade in Retinal I/R Injury

**Figure 3:**
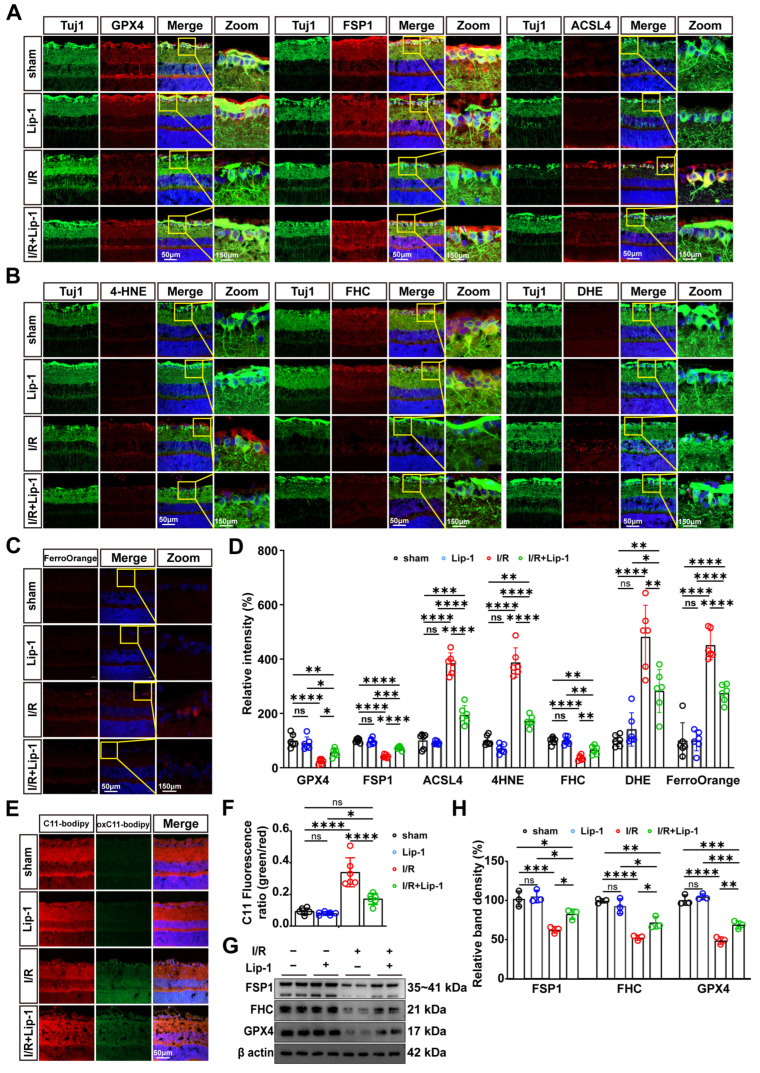
**Lip-1 halts the oxidative stress-to-ferroptosis cascade in retinal I/R injury.** (**A**) Immunofluorescence co-staining of Tuj1-positive RGCs (green) with GPX4 (red), FSP1 (red), and ACSL4 (red) and DAPI (blue) at 24 h post-I/R in retinal sections from sham and I/R mice treated with Vehicle or Lip-1. (**B**) Immunofluorescence co-staining of Tuj1-positive RGCs (green) with 4HNE (red), FHC (red), and DHE (red) and DAPI (blue) at 24 h post-I/R in retinal sections from sham and I/R mice treated with Vehicle or Lip-1. (**C**) Immunofluorescence co-staining of FerroOrange (red) with DAPI (blue) at 24 h post-I/R in retinal sections from sham and I/R mice treated with Vehicle or Lip-1. (**D**) Quantification of the relative immunofluorescence intensity of GPX4, FSP1, ACSL4, 4-HNE, FHC, DHE, and FerroOrange from (**A**–**C**) (*n* = 6 biologically independent experiments). (**E**) Immunofluorescence co-staining of C11-BODIPY (green/red) staining with DAPI (blue) at 24 h post-I/R in retinal sections from sham and I/R mice treated with Vehicle or Lip-1. (**F**) Quantification of the C11 fluorescence ratio (green/red) from (**E**) (*n* = 6 biologically independent experiments). (**G**) Western blot analysis of GPX4, FSP1, and FHC expression from sham and I/R mice treated with Vehicle or Lip-1. β-Actin served as a loading control. (**H**) Quantification of GPX4, FSP1, and FHC protein levels from (**E**) (*n* = 3 biologically independent experiments). Scale bars: 50 μm. Total magnification: 400×. Data were analyzed by two-way ANOVA with Tukey’s post hoc test. All data are shown as mean ± SEM. ns, not significant, * *p* < 0.05, ** *p* < 0.01, *** *p* < 0.001, **** *p* < 0.0001.

To investigate the impact of Lip-1 on the molecular cascade of oxidative stress and ferroptosis, we assessed key regulators of these pathways following I/R injury. IF analysis revealed that I/R injury significantly downregulated the expression of key antioxidant defense proteins GPX4 and FSP1 in retinal tissues (both *p* < 0.0001, vs. sham) (Figure 3A,D). Colocalization studies with Tuj1 confirmed that this depletion occurred predominantly within RGCs. Lip-1 pretreatment effectively restored GPX4 and FSP1 expression levels compared with the I/R group (*p* = 0.0318 and *p* < 0.0001, respectively) (Figure 3A,D). Conversely, I/R injury markedly upregulates ACSL4 and accumulation of 4-HNE (both *p* < 0.0001, vs. sham), effects that were significantly reversed by Lip-1 pretreatment within RGCs (both *p* < 0.0001, vs. I/R) (Figure 3A,B,D).

We next assessed whether Lip-1 modulates iron dyshomeostasis, a critical driver of ferroptotic cell death. I/R injury suppressed FHC (*p* < 0.0001, vs. sham) and triggered Fe^2+^ accumulation in RGC (*p* < 0.0001, vs. sham), both of which were attenuated by Lip-1 pretreatment (*p* = 0.0054 and *p* < 0.0001, respectively, vs. I/R) (Figure 3B–D).

Systematic evaluation of oxidative stress markers further confirmed the antioxidant efficacy of Lip-1. DHE staining revealed a marked increase in the proportion of DHE-positive RGCs following I/R (*p* < 0.0001 vs. sham), which was significantly reduced by Lip-1 pretreatment (*p* = 0.0015 vs. I/R) (Figure 3B,D). Similarly, oxidation of the lipid peroxidation sensor C11-BODIPY581/591 was elevated in cells of the GCL after I/R (*p* < 0.0001 vs. sham), reflected by a decreased green/red fluorescence ratio. Lip-1 pretreatment preserved the ratio, consistent with suppression of oxidative lipid damage (*p* < 0.0001, vs. I/R) (Figure 3E,F). WB analysis corroborated these IF findings, demonstrating that Lip-1 reversed I/R-induced downregulation of FSP1 and GPX4, and restored FHC expression (*p* = 0.0458, *p* = 0.0082, and *p* = 0.0300, respectively, vs. I/R) (Figure 3G,H).

### 3.4. Lip-1 Pretreatment Suppresses I/R-Induced Glial Cell Activation and Neuroinflammation

**Figure 4:**
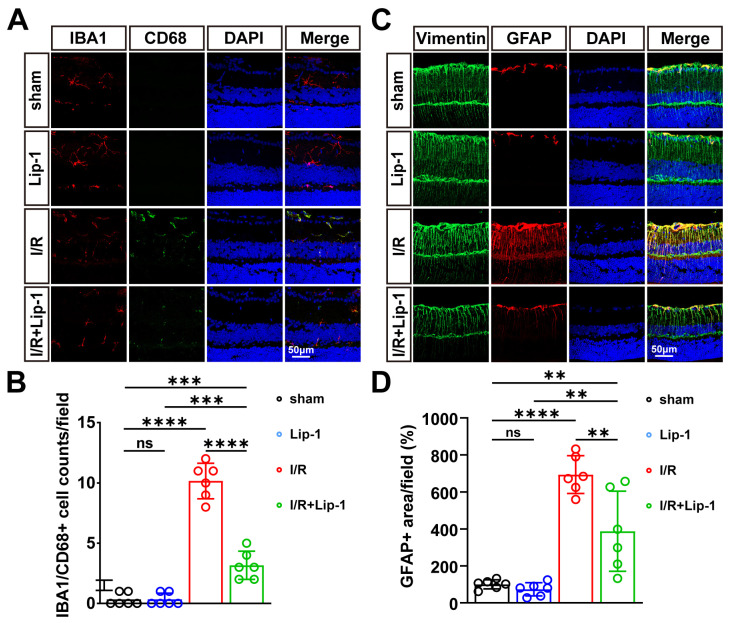
**Lip-1 Attenuates I/R-Induced Glial Reactivity and Neuroinflammation.** (**A**) Representative image of immunofluorescence of retinal macroglia: Iba1 (red), CD68 (green), DAPI (blue), and merged at 24 h post-I/R in retinal sections from sham and I/R mice treated with Vehicle or Lip-1. Scale bars: 50 μm. Images were acquired with a 40X objective lens. (**B**) Quantification of IBA1/CD68-positive cell counts/field from (**A**) (*n* = 6 biologically independent experiments). (**C**) Representative image of immunofluorescence of retinal macroglia: GFAP (astrocytes, red), Vimentin (Müller cells, green), DAPI (blue), and merges at 24 h post-I/R in retinal sections from sham and I/R mice treated with Vehicle or Lip-1. Scale bars: 50 μm. Total magnification: 400×. (**D**) Quantification of GFAP-positive area/field from (**C**) (*n* = 6 biologically independent experiments). Data were analyzed by two-way ANOVA with Tukey’s post hoc test. All data are shown as mean ± SEM. ns, not significant, ** *p* < 0.01, *** *p* < 0.001, **** *p* < 0.0001.

To investigate whether the neuroprotective effects of Lip-1 pretreatment extend to modulation of the neuroinflammatory milieu, we assessed glial cell responses following I/R injury with or without Lip-1 pretreatment. IF labeling for ionized calcium-binding adapter molecule 1 (Iba1), a microglial marker, revealed that in sham-treated retinas, microglia exhibited a resting, ramified morphology with fine branched processes and small somata, localized within INL and IPL (Figure 4A). Following I/R injury, microglia underwent robust activation, characterized by a marked increase in Iba1-positive cell density, migration toward the GCL, and a morphological shift toward an amoeboid phenotype with enlarged somata and retracted processes (Figure 4A). Co-labeling with cluster of differentiation 68 (CD68), a marker of phagocytic activity, confirmed that a substantial subset of these activated microglia acquired a phagocytic phenotype (Figure 4A). Quantification of Iba1/CD68 double-positive cells demonstrated a significant increase in the I/R group compared to sham controls (*p* < 0.0001) (Figure 4B). Lip-1 pretreatment profoundly attenuated microglial activation, as evidenced by a significant reduction in Iba1/CD68-positive cell counts (*p* < 0.0001 vs. I/R), preservation of ramified microglial morphology, and diminished migration into the GCL (Figure 4A,B).

We next examined the effect of Lip-1 pretreatment on macroglial reactivity. Immunostaining for glial fibrillary acidic protein (GFAP), an astrocyte activation marker, and vimentin, an intermediate filament protein upregulated in reactive Müller cells, revealed pronounced gliosis following I/R injury (Figure 4C). In sham retinas, GFAP immunoreactivity was largely confined to astrocytes in the GCL, with sparse labeling in Müller cell endfeet. Following I/R, GFAP expression intensified dramatically, with astrocytes extending thickened processes into the IPL, and Müller cells’ somata and radial processes becoming robustly GFAP-positive (Figure 4C). Quantitative analysis of the GFAP-positive area confirmed a significant increase following I/R (*p* < 0.0001 vs. sham), which was substantially reduced by Lip-1 pretreatment (*p* = 0.0016 vs. I/R).

### 3.5. Lip-1 Pretreatment Attenuates I/R-Induced Retinal Injury by Suppressing the EGR1/p53/xCT Pathway

**Figure 5:**
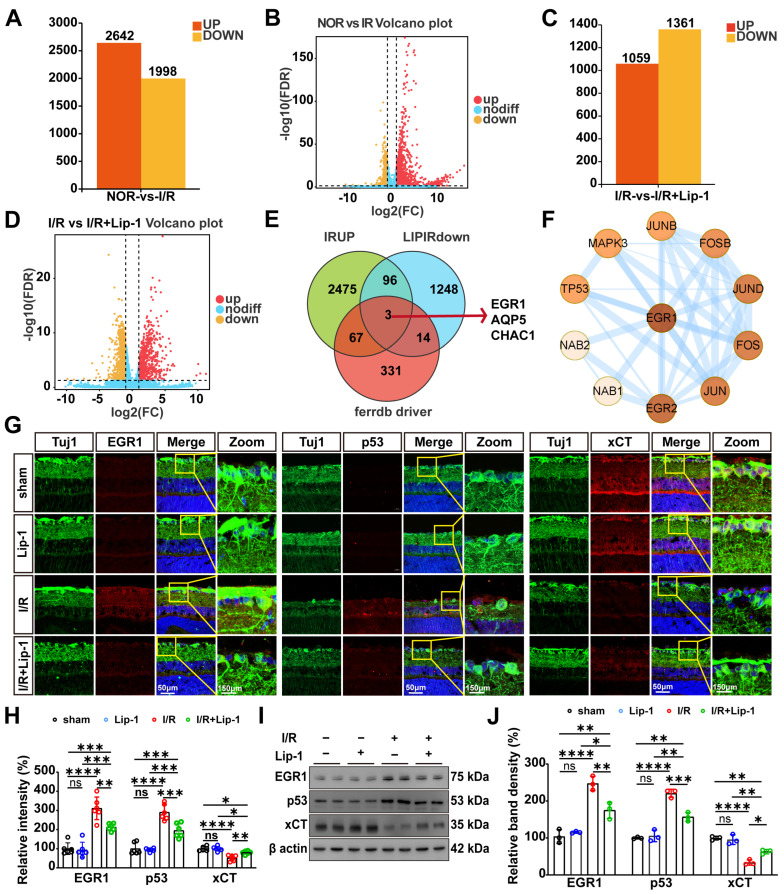
**EGR1/p53/xCT axis mediates Lip-1’s anti-ferroptotic effects in I/R-injured RGCs.** (**A**) Bar graph showing the number of differentially expressed genes (DEGs) identified in the comparison between the I/R + Vehicle group and the Sham group. Genes with |log2(Fold Change)| > 1.5 and an adjusted *p*-value < 0.05 were considered statistically significant. Upregulated genes are shown in red, and downregulated genes are shown in yellow. (**B**) Volcano plot from (**A**). (**C**) Bar graph showing the number of DEGs identified in the comparison between the I/R + Lip-1 group and the I/R + Vehicle group. Genes with |log2(Fold Change)| > 1.5 and an adjusted *p*-value < 0.05 were considered statistically significant. Upregulated genes are shown in red, and downregulated genes are shown in yellow. (**D**) Volcano plot from (**A**). (**E**) Venn diagram of FerrDb, upregulated genes from (**A**), and downregulated genes from (**C**). (**F**) The STRING interaction network of EGR1 (Mus musculus) (score > 0.9). (**G**) Immunofluorescence co-staining of Tuj1-positive RGCs (green) with EGR1 (red), p53 (red), xCT (red), and DAPI (blue) from sham and I/R mice treated with Vehicle or Lip-1. (**H**) Quantification of the relative immunofluorescence intensity of EGR1, p53, and xCT from (H) (*n* = 6 biologically independent experiments). Scale bars: 50 μm. Total magnification: 400×. (**I**) Western blot analysis of EGR1, p53, and xCT expression from sham and I/R mice treated with Vehicle or Lip-1. β-Actin served as a loading control. (**J**) Quantification of EGR1, p53, and xCT protein levels from (**G**) (*n* = 3 biologically independent experiments). Data were analyzed by two-way ANOVA with Tukey’s post hoc test. All data are shown as mean ± SEM. ns, not significant, * *p* < 0.05, ** *p* < 0.01, *** *p* < 0.001, **** *p* < 0.0001.

To elucidate the transcriptional mechanisms underlying Lip-1-mediated neuroprotection, we performed whole-transcriptome analysis of retinas from Sham, I/R, and I/R + Lip-1 groups. Comparative analysis revealed substantial transcriptional remodeling following I/R injury, with 4640 differentially expressed genes (DEGs) identified in I/R versus Sham controls (|log2FC| > 1.5, FDR < 0.05), comprising 2642 upregulated and 1998 downregulated transcripts (Figure 5A,B). Lip-1 pretreatment significantly modulated this injury-induced transcriptional landscape, as evidenced by 2420 DEGs in the I/R + Lip-1 versus I/R comparison (1059 upregulated, 1361 downregulated) (Figure 5C,D).

To identify ferroptosis-relevant transcriptional nodes targeted by Lip-1, we performed overlap analysis of three gene sets: Genes upregulated by I/R (I/R vs. sham), genes downregulated by Lip-1 pretreatment (I/R + Lip-1 vs. I/R), and ferroptosis-related genes cataloged in the FerrDb V2 database. This integrative approach yielded three candidate regulators: early growth response protein 1 (EGR1), aquaporin-5 (AQP5), and ChaC glutathione-specific γ-glutamylcyclotransferase 1 (CHAC1) (Figure 5E). We prioritized EGR1for further validation.

STRING protein-protein interaction analysis (v12.0; confidence score >0.7) predicted a high-confidence interaction (score > 0.9) between EGR1 and p53 (Figure 5F). Consistent with this prediction, I/R injury markedly upregulated EGR1 and p53 in RGCs (both *p* < 0.0001 vs. sham), as demonstrated by IF co-localization with Tuj1, and Lip-1 pretreatment effectively suppressed their induction specifically (*p* = 0.0020 and = 0.0002, respectively, vs. I/R) (Figure 5G,H).

We then assessed xCT expression, as p53 is a known transcriptional repressor of xCT. I/R injury led to significant downregulation of xCT immunoreactivity within RGCs (*p* < 0.0001 vs. sham). Lip-1 pretreatment partially but significantly restored xCT expression (*p* = 0.0031 vs. I/R) (Figure 5G–J). WB analysis of retinal lysates corroborated these IF findings, demonstrating that Lip-1 reversed I/R-induced upregulation of EGR1 and p53 while restoring xCT protein levels (*p* = 0.0040, *p* = 0.0006, and *p* = 0.0131, respectively, vs. I/R) (Figure 5I,J).

### 3.6. Lip-1 Pretreatment Suppresses OGD/R-Induced Ferroptosis in Primary RGCs by Restoring Antioxidant Defenses and Suppressing Lipid Peroxidation

**Figure 6:**
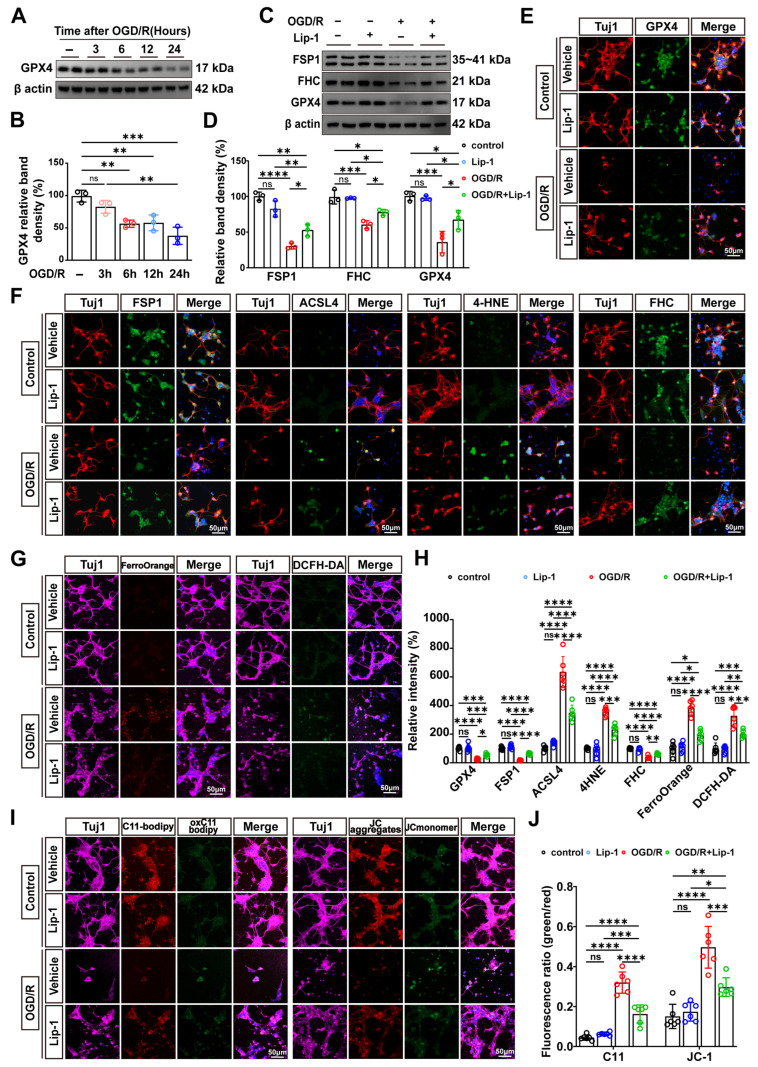
**Lip-1 attenuates OGD/R-induced ferroptosis in primary RGCs by restoring antioxidant defenses and suppressing lipid peroxidation.** (**A**) Western blot analysis of GPX4 expression in primary RGCs at the indicated time points (3, 6, 12, 24 h) after OGD/R. β-Actin served as a loading control. (**B**) Quantification of GPX4 relative band density from (**A**) (*n* = 3 biologically independent experiments). (**C**) Western blot analysis of GPX4, FSP1, and FHC expression in RGCs under different conditions (Control, OGD/R + Vehicle, OGD/R + Lip-1). β-Actin served as a loading control. (**D**) Quantification of GPX4, FSP1, and FHC protein levels from (**C**) (*n* = 3 biologically independent experiments). (**E**) Immunofluorescence co-staining of Tuj1-positive RGCs (red) with GPX4 (green) and DAPI (blue) under different conditions. (**F**) Immunofluorescence co-staining of Tuj1-positive RGCs (red) with ferroptosis markers (FSP1, ACSL4, 4-HNE, FHC, all in green) and DAPI (blue) under different conditions. (**G**) Immunofluorescence co-staining of Tuj1-positive RGCs (purple) with FerroOrange staining (red), DCFH-DA (green) and DAPI (blue) under different conditions. (**H**) Quantification of the relative immunofluorescence intensity of GPX4, FSP1, ACSL4, 4-HNE, FHC, FerroOrange, and DCFH-DA from (**E**–**G**) (*n* = 6 biologically independent experiments). (**I**) Immunofluorescence co-staining of Tuj1-positive RGCs (purple) with lipid peroxidation (detected by oxidized C11-BODIPY581/591, green/red), mitochondrial membrane potential (assessed by JC-1 monomer/aggregate ratio, green/red) and DAPI (blue) under different conditions. (**J**) Quantification of the C11 and JC-1 fluorescence ratio (green/red) from (**I**) (*n* = 6 biologically independent experiments). (**E**–**G**,**I**) Scale bars: 50 μm. Total magnification: 400×. Data were analyzed by two-way ANOVA with Tukey’s post hoc test. All data are shown as mean ± SEM. ns, not significant, * *p* < 0.05, ** *p* < 0.01, *** *p* < 0.001, **** *p* < 0.0001.

IF co-staining for Tuj1 and RBPMS confirmed successful primary RGC isolation (Figure S1A). To validate the ferroptosis-inhibitory mechanism of Lip-1 in a controlled cellular context, we established an OGD/R model using primary RGCs isolated from neonatal mice. We first monitored the expression of GPX4 during OGD/R. WB analysis revealed a progressive, time-dependent decline in GPX4 protein levels over the course of reperfusion, with maximal depletion observed at 24 h (*p* = 0.0002 vs. control) (Figure 6A,B).

Dose-response analysis demonstrated that Lip-1 pretreatment significantly improved cell survival over a broad concentration window, with protective effects plateauing between 100 nM and 500 nM (all *p* < 0.0001 vs. control) (Figure S1B). Based on these data, the intermediate dose of 200 nM was selected for subsequent experiments.

At the molecular level, Lip-1 pretreatment restored key components of the cellular antioxidant system. WB and IF analyses revealed Lip-1 pretreatment significantly reversed the OGD/R-induced downregulation of both FSP1 (WB, *p* = 0.0334; IF, *p* = 0.0146, both vs. OGD/R) and GPX4 (WB, *p* < 0.0001; IF, *p* = 0.0293, both vs. OGD/R) (Figure 6C–F,H). Concurrently, Lip-1 inhibited the OGD/R-induced upregulation of ACSL4 (*p* < 0.0001 vs. OGD/R) and markedly reduced the accumulation of 4-HNE (*p* < 0.0001 vs. OGD/R) (Figure 6F,H).

We next assessed whether Lip-1 modulates iron homeostasis in primary RGCs. OGD/R exposure resulted in robust accumulation of Fe^2+^ (*p* < 0.0001 vs. control), as detected by FerroOrange fluorescence, which was significantly attenuated by Lip-1 pretreatment (*p* < 0.0001 vs. OGD/R) (Figure 6G,H). Consistent with this finding, OGD/R-induced downregulation of FHC was partially but significantly restored by Lip-1 (WB, *p* = 0.0351; IF, *p* = 0.0018, both vs. OGD/R) (Figure 6C,D,F,H).

Lip-1 also effectively suppressed a broad spectrum of oxidative damage markers induced by OGD/R. The oxidation-sensitive probe C11-BODIPY581/591 revealed pronounced lipid peroxidation in RGCs following OGD/R (*p* < 0.0001 vs. control), reflected by a decreased green/red fluorescence ratio, which was significantly preserved by Lip-1 (*p* < 0.0001 vs. OGD/R) (Figure 6I,J). Mitochondrial membrane potential, assessed by JC-1 monomer-to-aggregate conversion, was depolarized after OGD/R (*p* < 0.0001 vs. control), whereas Lip-1 treatment partially restored JC-1 aggregate fluorescence (*p* = 0.0004 vs. OGD/R) (Figure 6I,J). General oxidative stress, measured by DCFH-DA fluorescence, was also markedly elevated following OGD/R (*p* < 0.0001 vs. control) and significantly reduced by Lip-1 (*p* = 0.0004 vs. OGD/R) (Figure 6G,H).

### 3.7. Lip-1 Pretreatment Inhibits OGD/R-Induced EGR1/p53/xCT Dysregulation in RGCs

**Figure 7:**
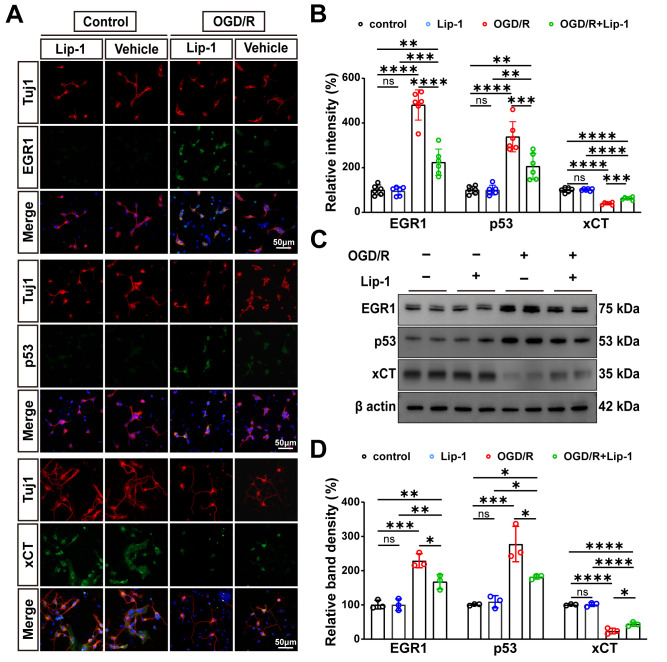
**Lip-1 Attenuates OGD/R-Induced EGR1/p53/xCT Dysregulation in RGCs.** (**A**) Immunofluorescence co-staining of Tuj1-positive RGCs (red) for EGR1 (green), p53 (green), xCT (green) and DAPI (blue) from control and OGD/R RGCs with vehicle or Lip-1. Scale bar: 50 μm. Total magnification: 400×. (**B**) Quantification of relative fluorescence intensity of EGR1, p53, and xCT from (**C**) (*n* = 6 biologically independent experiments). (**C**) Western blot analysis of EGR1, p53, and xCT expression from control and OGD/R RGCs with vehicle or Lip-1. β-actin served as a loading control. (**D**) Quantification of relative band density from (**A**) (*n* = 3 biologically independent experiments). Data were analyzed by two-way ANOVA with Tukey’s post hoc test. All data are shown as mean ± SEM. ns, not significant, * *p* < 0.05, ** *p* < 0.01, *** *p* < 0.001, **** *p* < 0.0001.

To validate whether Lip-1-mediated protection involves the transcriptional regulatory pathway identified in vivo, we subjected primary RGCs to OGD/R and assessed the expression of EGR1, p53, and xCT. Quantitative IF with Tuj1 revealed that OGD/R significantly elevated EGR1 and p53 while diminishing xCT in RGCs (all *p* < 0.0001 vs. control) (Figure 7A,B). Lip-1 pretreatment reversed these changes, reducing EGR1 and p53 upregulation (*p* < 0.0001 and *p* = 0.0005, respectively, vs. OGD/R) and partially restoring xCT expression (*p* = 0.0002 vs. OGD/R) (Figure 7A,B). WB analysis of RGC lysates confirmed these findings, demonstrating reduced EGR1 and p53 protein levels (*p* = 0.0134 and *p* = 0.0115, respectively) and increased xCT expression (*p* = 0.0159) in the OGD/R + Lip-1 group compared to OGD/R alone (Figure 7C,D).

### 3.8. EGR1 Knockdown Suppresses OGD/R-Induced Ferroptosis in RGCs by Inhibiting the EGR1/p53/xCT Pathway

**Figure 8:**
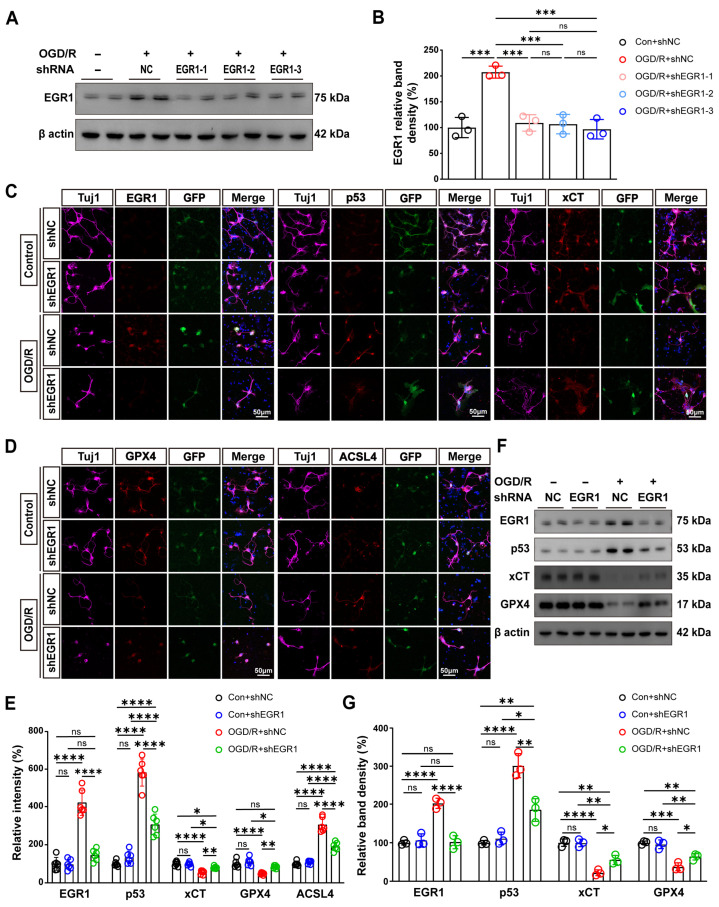
**EGR1 Knockdown Suppresses OGD/R-Induced Ferroptosis in RGCs by Suppressing the EGR1/p53/xCT Pathway.** (**A**) Western blot validation of EGR1 knockdown efficiency using three lentiviral shRNA constructs (shEGR1-1/2/3) in primary RGCs post-OGD/R, with shNC (non-targeting) as control. β-actin: loading control. (**B**) Quantification of EGR1 protein levels from (**A**) (*n* = 3 biologically independent experiments). (**C**) Immunofluorescence co-staining of Tuj1-positive RGCs (purple) for EGR1 (red), p53 (red), xCT (red), and DAPI (blue), with GFP (green) indicating lentiviral transduction.. Scale bar: 50 μm. Total magnification: 400×. (**D**) Immunofluorescence co-staining of Tuj1-positive RGCs (purple) for GPX4 (red), ACSL4 (red), and DAPI (blue), with GFP (green) indicating lentiviral transduction. Scale bar: 50 μm. Total magnification: 400×. (**E**) Quantification of relative fluorescence intensity of EGR1, p53, xCT, GPX4, and ACSL4 from (**C**,**D**) (*n* = 6 independent experiments). (**F**) Western blot analysis of EGR1, p53, xCT, and GPX4 expression from control and OGD/R RGCs with shNC or shEGR1. β-actin served as a loading control. (**G**) Quantification of EGR1, p53, xCT, and GPX4 protein levels from (**C**) (*n* = 3 biologically independent experiments). Data in panel (**B**) were analyzed by one-way ANOVA, and data in panels (**E**–**G**) by two-way ANOVA, both followed by Tukey’s post hoc test. ns, not significant, * *p* < 0.05, ** *p* < 0.01, *** *p* < 0.001, **** *p* < 0.0001.

To establish a causal relationship between EGR1 and the ferroptotic cascade observed in RGCs following OGD/R injury, we employed lentiviral shRNA-mediated EGR1 knockdown (shEGR1) in primary RGCs. WB analysis demonstrated that all three shEGR1 constructs achieved significant and comparable reduction of EGR1 protein levels under OGD/R conditions (all *p* < 0.001 vs. OGD/R + shNC), with no significant inter-construct differences (all *p* > 0.05) (Figure 8A,B). shEGR1-3 was selected in a blinded manner for all subsequent experiments to mitigate selection bias.

IF analysis of the four experimental groups revealed that under control conditions, RGCs exhibited low EGR1 and p53 immunoreactivity and high xCT expression (Figure 8C). In the OGD/R + shNC group, marked upregulation of EGR1 and p53 was observed (both *p* < 0.0001 vs. Con + shNC), accompanied by suppression of xCT (*p* < 0.0001 vs. Con + shNC) fluorescence intensity (Figure 8C,E). Critically, compared to OGD/R + shNC, EGR1 knockdown attenuated the OGD/R-induced upregulation of EGR1 and p53 (both *p* < 0.0001) while restoring xCT fluorescence intensity (*p* = 0.0011) (Figure 8C,F,G). WB analysis of RGC lysates corroborated these immunofluorescence findings, demonstrating that EGR1 knockdown decreased EGR1 and p53 protein levels (*p* < 0.0001 and *p* = 0.0013, respectively, vs. OGD/R + shNC) while restoring xCT expression following OGD/R (*p* = 0.0141 vs. OGD/R + shNC) (Figure 8F,G).

Examination of ferroptosis regulators demonstrated that OGD/R + shNC decreased GPX4 (WB, *p* = 0.0001; IF, *p* < 0.0001) and increased ACSL4 (*p* < 0.0001) expression compared to Con + shNC controls (Figure 8D–G). In the OGD/R + shEGR1 group, GPX4 expression was elevated (WB, *p* = 0.0307; IF, *p* = 0.0028) and ACSL4 expression was reduced (*p* < 0.0001) relative to OGD/R + shNC (Figure 8D–G).

## 4. Discussion

Retinal ischemia/reperfusion (I/R) injury—a central pathological event in glaucoma and retinal vascular occlusion—triggers oxidative stress that inexorably progresses to visual impairment and irreversible vision loss [30,34,35]. Current clinical management remains largely palliative, underscoring an urgent need for mechanism-anchored interventions [36,37]. The central finding of this study is the identification of the EGR1/p53 axis as a pivotal mechanistic hub in ferroptosis-driven retinal I/R injury. We demonstrate that the ferroptosis inhibitor Lip-1 confers robust neuroprotection by downregulating this signaling axis, preserving retinal structure and function, and that EGR1 knockdown phenocopies this effect—suppressing p53, restoring xCT, and positioning p53 downstream of EGR1 within a pro-oxidant transcriptional program.

EGR1 is an immediate-early gene rapidly induced by ischemia and reactive aldehydes such as 4-HNE, and has been implicated in neuropathic pain, inflammation, and sustained neuronal dysregulation through feed-forward oxidative loops [38,39,40]. As a master zinc-finger transcription factor governing cellular oxidative stress responses [41,42,43,44], EGR1 was robustly upregulated in RGCs following I/R and potently suppressed by Lip-1, consistent with its role as a conserved tissue stress marker [23,24,26,27,45,46]. Its sustained upregulation in RGCs after I/R injury—and its robust suppression by Lip-1—implicates EGR1 as a central amplifier of oxidative damage. Protein interaction analyses predicted a high-confidence association between the zinc-finger domain of EGR1 and the transactivation domain of p53. Functional validation confirmed that EGR1 knockdown reduces p53 expression and restores xCT, thereby inhibiting ferroptosis. This places p53 downstream of EGR1 in a pro-ferroptotic transcriptional cascade. While p53 is known to suppress xCT and promote ferroptosis in cerebral and renal I/R [23,47,48], its regulation by EGR1 in the ischemic retina has not, to our knowledge, been previously reported. p53 functions as a direct transcriptional repressor of xCT in the retina [23]; its suppression of xCT cripples cellular cystine uptake and glutathione biosynthesis—a critical event that creates a permissive environment for oxidative damage and ferroptosis. Whether this interaction reflects direct physical binding or requires additional co-factors awaits biochemical validation via co-immunoprecipitation or biophysical assays. Moreover, p53-mediated ferroptosis is frequently modulated by post-translational modifications such as Ser15 phosphorylation [48]; whether EGR1 influences p53 phosphorylation or Lip-1 intersects with this modification remains an open question. Beyond transcriptional control, lipid metabolic reprogramming critically shapes ferroptosis susceptibility [49]. The concurrent upregulation of ACSL4—a key enzyme that catalyzes the esterification of polyunsaturated fatty acids (PUFAs) to facilitate lipid peroxidation [50], and a transcriptional target of EGR1 [51]—and p53 activation observed in our I/R model may collaboratively remodel membrane lipid composition toward a peroxidation-prone state. Lip-1’s dual suppression of ACSL4 and p53 could therefore restore MUFA/PUFA homeostasis, a hypothesis that warrants direct testing through retinal lipidomics. Given that EGR1 is also upregulated in various ocular pathologies [51,52,53], this axis may represent a conserved vulnerability across species and disease etiologies.

A transcriptionally targeted mechanism distinguishes Lip-1 from conventional ferroptosis inhibitors. Unlike ferrostatin-1 or melatonin, which primarily function as direct radical scavengers or indirect antioxidants, Lip-1 exerts its protective effects in our model through suppression of the EGR1/p53/xCT signaling axis. This conclusion is supported by convergent pharmacological and genetic evidence: Lip-1 administration downregulated EGR1 and p53, restored xCT expression, and suppressed ACSL4; conversely, Egr1 knockdown phenocopied these effects and attenuated OGD/R-induced ferroptosis. However, Lip-1 is well-characterized as a radical-trapping antioxidant (RTA) rather than a classical transcriptional regulator [20]. Thus, the observed EGR1 suppression may occur secondarily to reduced oxidative stress rather than through direct transcriptional inhibition. Without comparator compounds such as Ferrostatin-1, NAC, or deferoxamine, we cannot formally exclude non-specific antioxidant effects. GPX4 serves as a central regulator, preventing lethal lipid peroxidation through the reduction of phospholipid hydroperoxides using glutathione [54,55], while FSP1 acts through a parallel, NADPH-dependent pathway for coenzyme Q10 reduction and radical trapping [56,57]. The restoration of both GPX4 and FSP1 by Lip-1, together with inhibition of ACSL4 and reduction of 4-HNE—a toxic end-product of lipid peroxidation [9]—indicates multi-level suppression of the ferroptotic cascade. Notably, Lip-1’s molecular conformation enables sustained activity within membranes, and its oxidation products retain RTA capacity—properties that may synergize with its transcriptional effects [20]. However, whether EGR1 suppression occurs secondary to redox normalization or via direct engagement by Lip-1 remains an open question that warrants structure-activity relationship studies and chromatin profiling. Collectively, these findings establish the EGR1/p53/xCT cascade as a therapeutically targetable node in oxidative neurodegeneration and advance Lip-1 beyond the prevailing view of ferroptosis inhibitors as purely radical-trapping antioxidants.

Beyond RGC protection, Lip-1 significantly preserved calretinin-positive amacrine cells and PKCα-positive bipolar cells, indicating a broader neuroprotective spectrum than previously appreciated. The preservation of these synaptic structures implies that Lip-1 alleviates oxidative damage to neuronal integrity, protecting inner retinal neurons, which share a common vulnerability to I/R-induced redox imbalance owing to their high metabolic demand and synaptic activity. A notable finding of this study is that Lip-1 potently attenuates both microgliosis and astrogliosis in the I/R-injured retina—an effect achieved at the same dose regimen conferring neuroprotection. Reactive gliosis is a major source of secondary inflammatory oxidants, and its suppression by Lip-1 likely contributes to the overall neuroprotective outcome. This dual action is functionally significant because reactive glia are not merely passive responders but active amplifiers of oxidative injury, generating NADPH oxidase–derived ROS and pro-inflammatory cytokines (e.g., IL-1β, TNF-α) that perpetuate redox imbalance and secondary neurodegeneration [58,59]. By suppressing glial activation, Lip-1 disrupts this self-amplifying cycle at its origin. These findings align with reports of Lip-1 limiting inflammatory infiltration in renal and spinal cord I/R models [23,60], positioning the compound as a broad-spectrum inhibitor of sterile inflammation driven by oxidative stress. Whether its anti-gliotic effects in the retina reflect direct modulation of glial signaling, indirect preservation of neuronal redox homeostasis, or both remains to be determined and will require cell-type-specific genetic dissection.

Lip-1 is known to exert pleiotropic effects beyond canonical ferroptosis inhibition, including modulation of vitamin K metabolism through VKORC1L1 [61] and suppression of apoptosis, pyroptosis, or necroptosis in diverse extraocular models [62,63,64,65,66]. However, these pathways were not directly tested in our current study, and their contribution to the protection observed here remains speculative. Therefore, the most parsimonious explanation for Lip-1’s efficacy in retinal I/R injury remains its well-established anti-ferroptotic activity together with our newly identified suppression of the EGR1/p53/xCT axis. This mechanistic delineation positions the transcriptional modulation of stress-responsive factors as a viable neuroprotective strategy.

A key translational consideration is Lip-1’s physicochemical profile. As a spirocyclic piperidine derivative, Lip-1 is highly lipophilic and integrates efficiently into lipid bilayers, where it intercepts propagating lipid radicals via hydrogen atom donation [20]. Its molecular structure enables sustained radical-trapping activity, with oxidation products retaining efficacy and achieving higher stoichiometric potency than conventional antioxidants [20]. Beyond its established role as a radical-trapping antioxidant, Lip-1 can chelate iron via reversible coordination complexes with Fe^3+^ through its piperidine, imine, and aniline groups [67]. Upregulation of FHC enhances iron sequestration, thereby limiting the labile iron pool available for Fenton chemistry [16,68,69]. However, this same lipophilicity poses challenges for ocular delivery. Systemic administration of unmodified Lip-1 may achieve therapeutic concentrations in highly perfused tissues, but its retinal bioavailability is limited by the blood–retinal barrier. Intravitreal injection—the approach adopted in this study and a clinically established route for retinal neuroprotectives—offers a direct means to bypass systemic bioavailability limitations and achieve therapeutic concentrations within the vitreous and retina. This approach enables localized delivery with reduced systemic exposure, though it carries inherent drawbacks, including procedural invasiveness, risk of endophthalmitis, cataractogenesis, and the need for repeated injections in chronic disease contexts. These limitations underscore the potential utility of sustained-release formulations; nanoparticle-based carriers, hydrogels, and intraocular implants have been shown to enhance Lip-1 efficacy at lower doses in non-ocular systems [70,71] and warrant evaluation in the eye.

Beyond these delivery considerations, the lipophilic nature of Lip-1 also raises a fundamental question regarding its mechanism of action: whether its effects are confined exclusively to the plasma membrane. While our present study did not directly investigate its subcellular localization, our functional data provide indirect evidence supporting an intracellular site of action. We observed that Lip-1 treatment significantly mitigated the reduction in intracellular Fe^2+^ levels induced by I/R or OGD/R injury. This observation aligns with the well-documented lysosomotropic properties of Lip-1, which facilitate its accumulation within CD44^+^ Müller glia, where it can chelate labile iron and disrupt Fenton chemistry [67]. Therefore, the neuroprotective effects of Lip-1 are likely not restricted to radical scavenging at the plasma membrane but also involve critical intracellular events, particularly the regulation of labile iron pools within lysosomes. This dual-mode of action may underlie the biphasic dose-response observed in our study—optimal protection at 200 nM, with diminished efficacy above 500 nM. Higher concentrations (>500 nM) may approach solubility limits or elicit cytotoxic effects often associated with lipophilic compounds, potentially reflecting a pathological shift from beneficial membrane radical scavenging to excessive, potentially deleterious, lysosomal iron sequestration. Consequently, precision in both dose optimization and delivery platform design will be critical for the successful clinical translation of Lip-1 as a neuroprotective agent for retinal disease.

Several limitations of this study should be acknowledged. First, as Lip-1 is primarily an RTA rather than a transcriptional regulator [20], the observed EGR1 suppression may reflect secondary effects of ROS reduction. Without controls such as Fer-1, NAC, or deferoxamine, non-specific antioxidant effects cannot be excluded. Second, the predicted physical interaction between EGR1 and p53, while strongly supported by functional data, requires direct biochemical confirmation via co-immunoprecipitation or surface plasmon resonance. Third, although our data most parsimoniously support ferroptosis as the primary execution pathway, we cannot formally exclude contributions from other Lip-1-sensitive death modalities (e.g., apoptosis, necroptosis) under our experimental conditions; these were not directly tested and remain open to investigation. Fourth, the mechanistic basis of Lip-1’s biphasic dose-response, while hypothesized to involve lysosomal iron sequestration, awaits direct measurement of labile iron pools and lipid peroxidation at supra-optimal concentrations. Finally, the acute murine I/R model, while mechanistically tractable, does not fully recapitulate the chronic, progressive nature of glaucomatous neurodegeneration. Validation in chronic ocular hypertension models, assessment of long-term visual function, and exploration of EGR1 as a broader therapeutic entry point for other redox-driven retinal disorders are essential next steps.

## 5. Conclusions

In conclusion, this study identifies EGR1 as a previously unrecognized transcriptional driver of ferroptosis in retinal ischemia–reperfusion injury and positions Lip-1 as the first pharmacological agent, to our knowledge, capable of disrupting this pathogenic axis via modulation of the EGR1/p53/xCT pathway. Rather than functioning solely as a broad-spectrum radical-trapping antioxidant, Lip-1 engages a specific, therapeutically targetable transcription program—a conceptual advance that moves beyond the prevailing view of ferroptosis inhibitors as mere redox scavengers. By demonstrating that genetic Egr1 silencing phenocopies Lip-1–mediated protection, we establish EGR1 as a mechanistic linchpin and provide proof-of-concept that upstream transcription factor modulation can achieve neuroprotection equivalent to direct ferroptosis inhibition. These findings hold translational relevance beyond acute I/R injury: Lip-1 is orally bioavailable, exhibits favorable pharmacokinetics, and has already shown efficacy in non-ocular I/R models, collectively supporting its repurposing potential for chronic glaucomatous neurodegeneration. Moreover, the convergence of Lip-1–sensitive transcriptional nodes with ferroptosis signatures observed in human glaucomatous retinas suggests that targeting EGR1-governed networks may offer a disease-modifying strategy. Future efforts should prioritize validation in chronic ocular hypertension models, assessment of long-term visual function, and exploration of EGR1 as a broader therapeutic entry point for other redox-driven retinal disorders.

## Figures and Tables

**Table 1:** Antibody list.

Antibody	Host Species	Company	Catalog Number	Dilution/Concentration
ACSL4	Rabbit	Abcam	ab155282	1:200 (IF)
Calretinin	Rabbit	Abcam	ab702	1:200 (IF)
Calbindin	Mouse	Abcam	ab82812	1:200 (IF)
CD68	Rat	BIO-RAD	MCA1957	1:200 (IF)
EGR1	Rabbit	Abcam	ab300449	1:200 (IF); 1:500 (WB)
FSP1	Rabbit	Proteintech	20886-AP	1:1000 (WB)
FSP1	Rabbit	Bioss	bs-7655R	1:200 (IF)
FHC	Rabbit	Bioss BIOLOGICALS	bs-8679R	1:200 (IF,); 1:1000 (WB)
GPX4	Mouse	Proteintech	67763	1:200 (IF)
GPX4	Rabbit	Abways	CY6959	1:1000 (WB)
GFAP, Cy3	Mouse	MILLIPORE	MAB340	1:200 (IF)
Iba-1	Rabbit	Wako	019-19741	1:200 (IF)
FHC	Rabbit	Bioss BIOLOGICALS	bs-8679R	1:200 (IF,); 1:1000 (WB)
xCT	Mouse	NOVUS	NB300-318SS	1:200 (IF)
xCT	Rabbit	Abcam	ab216876	1:1000 (WB)
Iba-1	Rabbit	Wako	019-19741	1:200 (IF)
CD68	Rat	BIO-RAD	MCA1957	1:200 (IF)
Vimentin	Mouse	Servicebio	GB12192	1:200 (IF)
GFAP, Cy3	Mouse	MILLIPORE	MAB340	1:200 (IF)
PKC-α	Mouse	Santa Cruz	sc-8393	1:200 (IF)
P53	Mouse	NOVUS	NBP2-294538	1:200 (IF)
P53	Rabbit	Bioss	BS-8687R	1:1000 (WB)
RBPMS	Rabbit	GeneTex	GTX118619	1:200 (IF)
RBPMS	Rabbit	MILLIPORE	ABN1362	1:200 (IF)
xCT	Mouse	NOVUS	NB300-318SS	1:200 (IF)
xCT	Rabbit	Abcam	ab216876	1:1000 (WB)
Tuj1	Rabbit	BioLegend	802001	1:500 (IF)
Tuj1	Mouse	BioLegend	801202	1:500 (IF)
Vimentin	Mouse	Servicebio	GB12192	1:200 (IF)
4-HNE	Mouse	Invitrogen	MA5-27570	1:50 (IF)

## Data Availability

RNA-seq data in this study have been deposited in the Sequence Read Archive of NCBI with the accession code PRJNA1393278. Further inquiries can be directed to the corresponding authors.

## Supplementary Materials

The following supporting information can be downloaded at: https://www.mdpi.com/article/10.3390/antiox15030391/s1. Supplementary Figure S1. Validation of purified RGCs and the concentration-dependent protective effect of Lip-1 against OGD/R injury.

## Abbreviations

The following abbreviations are used in this manuscript:

AQP5	aquaporin-5
CHAC1	ChaC glutathione-specific γ-glutamylcyclotransferase 1
DEGs	differentially expressed genes
DHE	dihydroethidium
DMSO	dimethyl sulfoxide
ERG	electroretinography
EGR1	early growth response 1
Fer-1	ferrostatin-1
GCC	ganglion cell complex
GPX4	glutathione peroxidase 4
H&E	Hematoxylin and Eosin
I/R	ischemia–reperfusion
IPL	inner plexiform layer
INL	inner nuclear layer
Lip-1	liproxstatin-1
NFL/GCL	nerve fiber layer/ganglion cell layer
OGD/R	oxygen–glucose deprivation/reoxygenation
ONL	outer nuclear layer
OPL	outer plexiform layer
OPs	oscillatory potentials
PhNR	photopic negative response
RBPMS	RNA-binding protein with multiple splicing
RGC	retinal ganglion cell
RNFL	retinal nerve fiber layer
SD-OCT	spectral-domain optical coherence tomography
shEgr1	Lentivirus-carrying Egr1-targeting shRNA
shNC	Lentivirus-carrying non-targeting control shRNA
Tuj1	neuron-specific class III beta-tubuli
xCT	solute carrier family 7 member 11
