# Supplementary material for: Liproxstatin-1 Attenuates Retinal Ischemia–Reperfusion Injury by Suppressing EGR1-Mediated Ferroptosis
Source: Antioxidants (Basel). 2026 Mar 19;15(3):391. doi: 10.3390/antiox15030391 (PMC13024618; doi:10.3390/antiox15030391)
Supplement: Supplementary file 1 [file antioxidants-15-00391-s001.zip › antioxidants-4183857-supplementary.pdf]

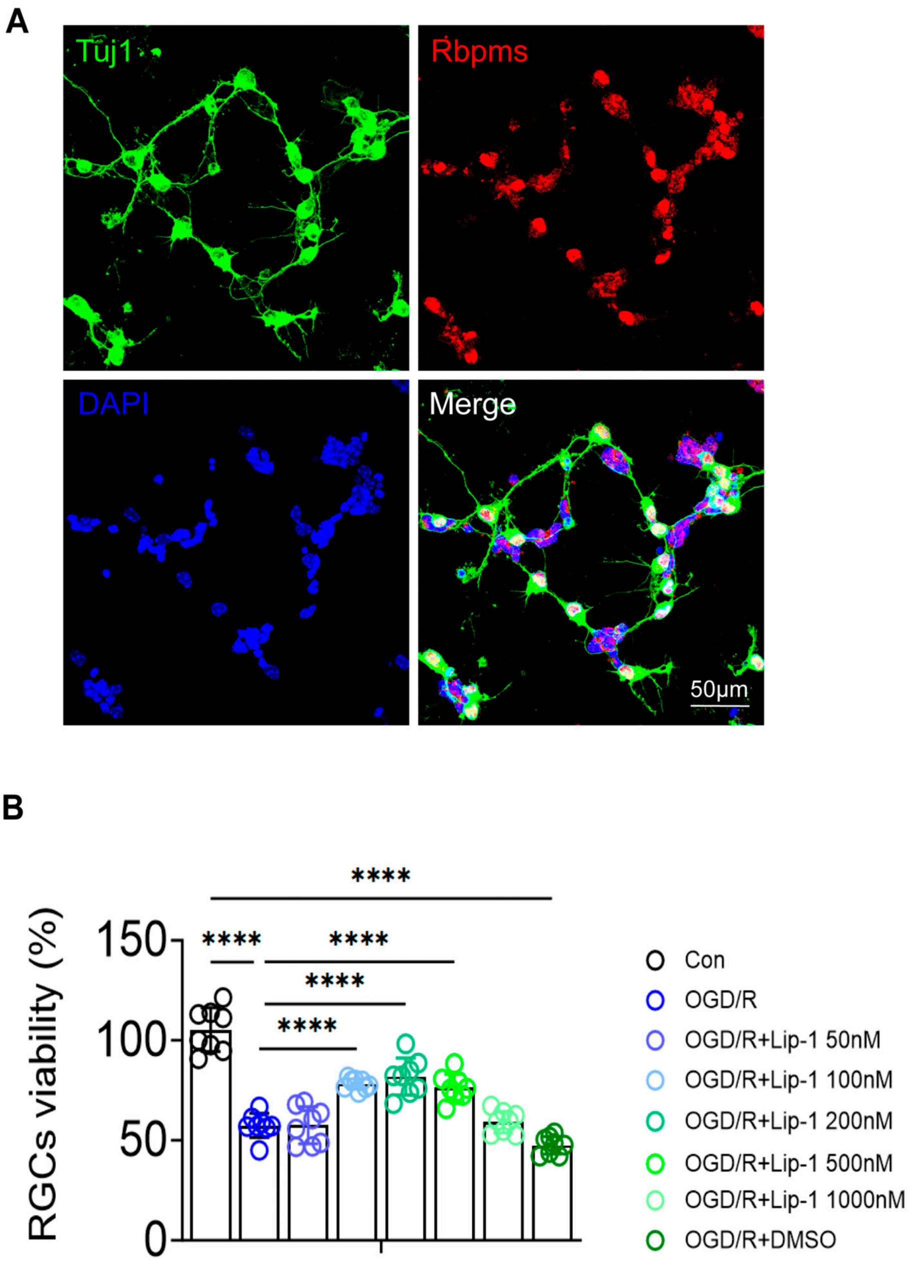

Figure S1. Validation of purified RGCs and the concentration-dependent protective effect of Lip-1 against OGD/R injury. (A) Immunofluorescence staining of primary RGCs purified from mouse retina. Cells were co-stained for the pan-neuronal marker neuron-specific class III beta-tubulin (Tuj1, red) and the RGC-specific marker RNA-binding protein with multiple splicing (RBPMS, green). Nuclei were counterstained with DAPI (blue). The merge image demonstrates a high degree of colocalization, confirming the high purity and successful isolation of the RGC culture. Scale bar: 50 μm. Total magnification: 400X. **(B)** Dose-response analysis of Lip-1 on RGC viability following OGD/R injury. Purified RGCs were subjected to OGD/R and treated with increasing concentrations of Lip-1 (50 to 1000 nM) or an equivalent volume of vehicle control (DMSO). Cell viability was significantly reduced after OGD/R compared to the control group. Lip-1 treatment conferred a concentration-dependent protective effect, with maximal efficacy observed at 200 nM. Data were analyzed by one-way ANOVA with Tukey's post hoc test. All data are shown as mean ± SEM. ns, not significant, ****$p < 0.0001$.
